# Micronucleus test using formalin-fixed rat glandular stomach and colon

**DOI:** 10.1186/s41021-023-00259-4

**Published:** 2023-01-14

**Authors:** Wakako Ohyama, Yohei Fujiishi, Emiko Okada, Kazunori Narumi, Makoto Hayashi

**Affiliations:** 1 Yakult Central Institute, Yakult Honsha Co., Ltd., 5-11 Izumi, Kunitachi-shi, Tokyo, 186-8650 Japan; 2 makoto international consulting, 4-23-3-1, Kamiimaizumi, Ebina-shi, Kanagawa 243-0431 Japan

**Keywords:** Micronucleus test, Gastrointestinal tract, Glandular stomach, Colon, Formalin-fixation method

## Abstract

**Background:**

Genotoxicity in tissues other than hematopoietic tissues, such as the liver and gastrointestinal (GI) tract, is an important focus in the risk assessment of chemicals in humans. We previously developed a rat micronucleus test for the GI tract, which is the first contact tissue where chemicals are introduced into the body through oral exposure. Target cells were obtained from fresh tissue samples by ethylenediaminetetraacetic acid disodium salt (EDTA) treatment. As an improvement to this method, we have used formalin-fixed tissues instead of fresh tissues; this approach can be used for tissues that are sampled from other toxicological tests and that are archived for several years. This new method can be used for examining micronucleus induction retrospectively when needed. In the present study, we compared the performance of the EDTA method and the new method with formalin-fixed tissues (formalin-fixation method).

**Results:**

Histological examination showed that both the EDTA and formalin-fixation methods could be used for collecting cells located in or above the proliferative zone of the GI tract tissues of rats. In addition, the collected cells were similar in shape. We conducted micronucleus tests with rat GI tract tissues by the two methods using model chemicals, which were used as positive control chemicals (a combination of diethylnitrosamine, 1,2-dimethylhydrazine dihydrochloride, and potassium bromate). The two methods showed similar results. We additionally evaluated the aging effect of tissues stored in formalin fixative. The results showed that 1 year of storage did not affect the frequency of micronucleated cells.

**Conclusion:**

The equivalence of the EDTA and formalin-fixation methods was confirmed, and micronucleus analysis was possible up to at least 1 year after formalin fixation of the GI tract, indicating that the formalin-fixation method is valuable for the rat GI tract micronucleus test.

## Introduction

Evaluation of genotoxicity in tissues other than hematopoietic tissue, such as the liver and gastrointestinal (GI) tract, is important in the risk assessment of chemicals in humans, particularly in in situ tissues that are the targets for carcinogenesis. Furthermore, the GI tract is the first contact site for orally exposed substances. DNA damage and gene mutations can be evaluated using appropriate tools such as the comet assay and gene mutation assay in transgenic animals [1, 2]. These assays can be performed on any tissue. On the other hand, the endpoint of chromosomal aberrations can be detected only in limited tissues. Recently, micronucleus (MN) assays using the skin, liver, GI tract, and germ cells have been developed and reviewed in several reports [3–7]. In particular, liver MN assays have undergone rapid advancements [6, 8, 9]. We previously developed a rat GI tract MN test by repeated treatment, and target cells were collected by ethylenediaminetetraacetic acid disodium salt (EDTA) treatment immediately after necropsy (EDTA method) [10–13]. This method showed good sensitivity and specificity for the detection of chemicals that induce chromosomal aberrations in the stomach and colon [8, 12–14]. As an improvement to the previous EDTA method, we have used formalin-fixed tissues instead of fresh tissues, on the basis of published reports [9, 15]; this approach can be used for tissues that are sampled from other toxicological tests and that are archived for several years. Using this new method (formalin-fixation method, abbrev.; FF method), we can examine MN induction retrospectively when needed without any additional experiment using new animals.

In the present study, we evaluated the performance of the MN assay with model chemicals by comparing the EDTA method with the new FF method using formalin-fixed tissues. Since the toxicological target organ is unknown in many cases, we assumed to conduct the MN tests on multiple tissues such as the GI tract, liver and bone marrow in a 2- or 4-week period study. As the model chemicals, we used a combination of three chemicals, diethylnitrosamine (DEN), 1,2-dimethylhydrazine dihydrochloride (DMH), and potassium bromate (KBrO_3_), because DEN gave positive results in the liver, DMH in the colon, and KBrO_3_ in the stomach and bone marrow after one or more administrations [10–12, 16]. The treatment regimen was the same as previously reported [12], except for one of the chemicals, KBrO_3_. In the previous treatment regimen with a single dose of DEN 15 days before necropsy, a single dose of DMH 4 days before necropsy, and double doses of *N*-nitroso-*N*-methylurea (MNU) 2 and 1 days before necropsy, significant increases in the frequencies of MNed cells were shown in all four tissues, but severe cytotoxicity in the bone marrow was observed. To mitigate the cytotoxicity, MNU was replaced with KBrO_3_ in the present study.

We also evaluated the aging effect of tissues stored in formalin fixative.

## Materials and methods

### Animals

Male Crl:CD (SD) rats were purchased from The Jackson Laboratory Japan Inc. (Kanagawa, Japan). The rats were housed together in groups of two or three in each cage with wood chip bedding at 23 ± 3 °C and 30–70% humidity with alternating 12 h intervals of light and dark. Animals were acclimatized for a week or more and treated at 8 weeks of age. All animals were fed F-2 commercial pellets (Funabashi Farm Co. Ltd., Chiba, Japan) and tap water ad libitum throughout the acclimation and experimental period. All experiments were performed in accordance with the guidelines for the care and use of laboratory animals established by the Institutional Animal Care and Use Committee of Yakult Central Institute, and the protocols were approved by this committee.

### Chemicals

DEN [55–18-5] (99.9% purity, Tokyo Chemical Industry Co., Ltd., Tokyo, Japan), DMH [306–37-6] (100% purity, Tokyo Chemical Industry Co., Ltd.), and KBrO_3_ [7758-01-2] (≥99.8% purity, FUJIFILM Wako Pure Chemical Corp., Osaka, Japan) were dissolved in water for injection (DW; Otsuka Pharmaceutical Factory, Inc., Tokushima, Japan) immediately before treatment. Ten% neutral-buffered formalin (Mildform® 10 N, which contains 1 w/w% methanol, FUJIFILM Wako Pure Chemical Corp.), which is routinely used in histopathological studies, was used to fix the cells and tissues used in the present study. Acridine orange (AO; FUJIFILM Wako Pure Chemical Corp.) and/or 4′,6-diamidino-2-phenylindole dihydrochloride (DAPI; Sigma Aldrich Co. LLC, St. Louis, USA) were used to stain the cells for MN analysis. Potassium hydroxide (KOH; FUJIFILM Wako Pure Chemical Corp.) was used to loosen the cell connections and prepare single-cell suspensions. Tris base and Tris hydrochloride (Sigma Aldrich Co. LLC) were used for preparing a Tris-HCl buffer.

### Micronucleus test

**Fig. 1:**
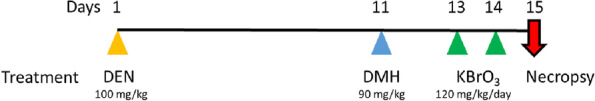
Experimental schedule. The animals in the treatment group were administered DEN (100 mg/kg) on Day 1, DMH (90 mg/kg) on Day 11, and KBrO_3_ (120 mg/kg/day) on Day 13 and 14 by oral gavage. Untreated animals were used for the negative controls

Five rats were randomly assigned to either the negative (untreated) control or the treatment group. The dose levels for the treatment group were set at 100 mg/kg body weight for DEN (a single dose), and 90 mg/kg body weight for DMH (a single dose) based on our previous report [12]. The dose level of KBrO_3_ was set at 120 mg/kg body weight/day (2 doses), showing positive results in the stomach and bone marrow with weak cytotoxicity, based on the results of a 2-week dose-finding study of KBrO_3_ when rats were administered a combination of DEN, DMH and KBrO_3_ with the same schedule as the present study. The treatment volume of each chemical was 10 mL/kg body weight. Each formulation was administered to the rats by oral gavage immediately after preparation. The treatment protocol is shown in Fig. 1. Day 1 is designated as the first day of treatment. On the day of necropsy (Day 15), animals were anesthetized with isoflurane and euthanized by exsanguination via the abdominal aorta, and their stomachs, colons, livers, and right femurs were sampled. The MN tests were conducted twice. Experiment 1 (Exp.1) was conducted to compare the performance between the EDTA and FF methods using the GI tract tissues obtained from the same animal; half of the glandular stomach and a portion of the colon were subjected to the EDTA method immediately after dissection from animals, and the remaining portion of these tissues were immersed in 10% neutral-buffered formalin to apply the FF method. Experiment 2 (Exp.2) was conducted to evaluate the aging effect of tissues stored in formalin fixative, comparing tissues from the same animal stored in formalin fixative for approximately 10 days (initial) and 1 year.

#### EDTA method (GI tract)

Single-cell preparation from fresh GI tract tissues using the EDTA method was performed according to our previous report [10]. The cell suspensions were washed, fixed in 10% neutral-buffered formalin, and stored at 4 °C until analysis. Immediately before microscopic observation, the cell suspensions were mixed with an equal volume of staining solution (250 μg/mL AO–2.5 μg/mL DAPI for the stomach and 500 μg/mL AO–2.5 μg/mL DAPI for the colon) on a glass slide. The cells were observed under a fluorescence microscope (600× magnification) with UV excitation (365 nm).

#### FF method (GI tract)

The entire stomach and colon were dissected from the rats and immersed in 10% neutral-buffered formalin for a week or more. After washing with DW, the fixed stomach and middle portion (approximately 3 cm long) of the colon were cut open along the greater curvature and longitudinally, respectively, and rinsed with DW. Subsequently, the forestomach was removed, and half of the glandular stomach and opened colon were rinsed with DW again and separately placed into a centrifuge tube containing approximately 10 mL of aqueous solution of KOH. As a result of examining various combinations of the concentrations (0.5, 1, and 4 M) and treatment periods (5, 16, and 24 h) of KOH, suitable cells for MN analysis were obtained when treated with 1 M KOH for 24 h at room temperature, and these treatment conditions were used thereafter. After treatment with KOH, the tissues were rinsed with 0.5 M Tris-HCl buffer (pH 7.5) for neutralization. The epithelial cells of each tissue were scraped with a Cell Scraper S made of silicon rubber (10 mm width, Sumitomo Bakelite Co., Ltd., Tokyo, Japan). The cells were separated by pipetting 10 times with a Pasteur pipette and transferred to a centrifuge tube. The cell suspension was centrifuged at 200×*g* for 5 min, the supernatant was removed, and the cells were resuspended in Tris-HCl buffer. This step was repeated. Finally, the cells were resuspended in a small amount of the same buffer and stored at 4 °C until analysis. The protocol for observing the cells was the same as that used in the EDTA method except for the concentration of the staining solution: 200 μg/mL AO–15 μg/mL DAPI was used after optimization to distinguish between the nucleus and cytoplasm.

#### Liver and bone marrow

Slides were prepared from formalin-fixed livers for MN analysis, according to the method reported by Shigano et al. [9] with minor modification in the KOH concentration (1 M), washing buffer (0.5 M Tris-HCl buffer, pH 7.5), staining solution (25 μg/mL DAPI), and excitation wavelength (365 nm) for a fluorescence microscope. The bone marrow (BM) cells were collected by washing the femur cavity with 1 mL of 10% neutral-buffered formalin, stained with 40 μg/mL AO solution, and observed under a fluorescence microscope (600× magnification) with blue excitation (490 nm) [17].

#### MN analysis

For the stomach, colon, and liver, 2000 cells were analyzed per animal for determining the frequency of the MNed cells. For the BM, 2000 immature erythrocytes (IMEs) from each rat were analyzed for determining the frequency of the MNed IMEs, and more than 500 erythrocytes were analyzed for determining the percentage of IMEs among the total erythrocytes (%IME).

### Histological examination

Histological examination of the glandular stomach and colon was performed before and after cell isolation of untreated 8-week-old male rats. For the EDTA method, portions of the glandular stomach and colon were fixed in 10% neutral-buffered formalin and used as histological specimens before cell isolation. The remaining portions were treated with EDTA as described in the section of EDTA method (GI tract), and the tissues after treatment were also fixed in 10% neutral-buffered formalin and used as histological specimens after cell isolation. For the FF method, the entire stomach and colon obtained from another animal were fixed in 10% neutral-buffered formalin. Portions of the glandular stomach and colon were used as histological specimens before cell isolation, and the remaining portions were treated with KOH, as described in the section of FF method (GI tract) and used as histological specimens after cell isolation. Each sample was then embedded in paraffin and sectioned. The sections were stained with hematoxylin–eosin and observed under a light microscope.

### Statistical analysis

The statistical significance between the values obtained with the EDTA and FF methods, between the initial values and values 1 year after formalin fixation, and between %IME values of the negative control and treatment groups was analyzed by Student’s *t*-test (significance level of 0.05) after confirming the homogeneity of variance using BellCurve for Excel version 3.20 (Social Survey Research Information Co., Ltd., Tokyo, Japan). Differences in MNed cell frequency between the negative control and treatment groups were analyzed using Kastenbaum and Bowman’s tables with a significance level of 0.01 [18].

## Results

**Fig. 2:**
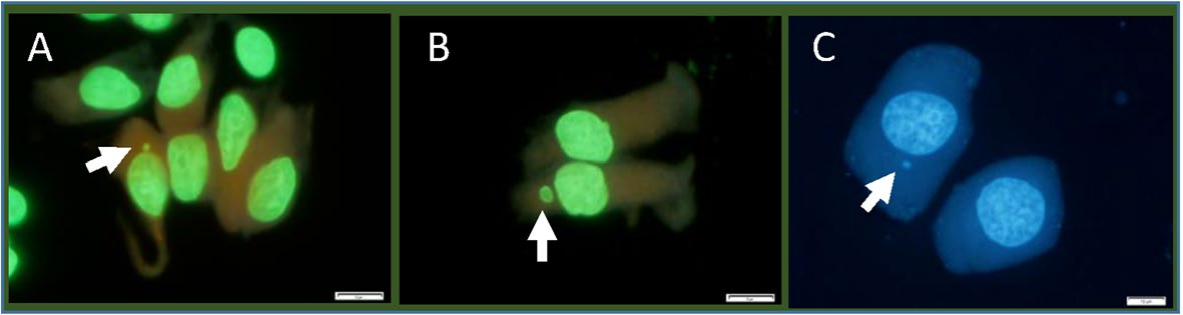
Representative images of MNed cells in the formalin-fixed tissues of rats in the treatment group. **A**, glandular stomach; **B**, colon; **C**, liver. Each arrow indicates a micronucleus. Each bar represents 10 μm

**Fig. 3:**
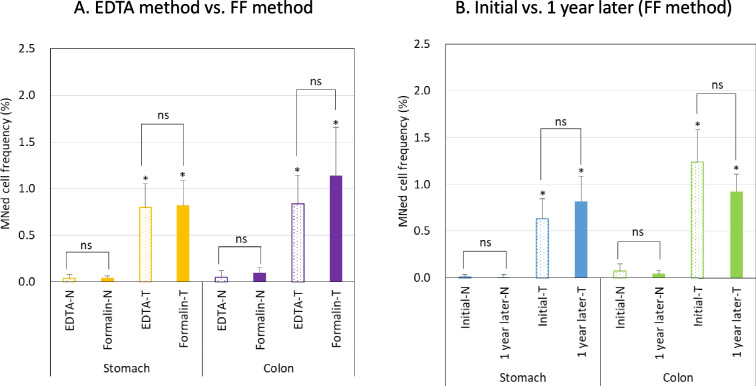
Comparison of MNed cell frequencies between the two methods **A** and between storage periods **B**. Each bar represents the frequency of MNed cells in the glandular stomach and colon (mean ± SD). N, negative control group; T, treatment group (combination treatment with DEN, DMH, and KBrO_3_). Statistical significance: ns, not significant (Student’s t-test); *P < 0.01 (Kastenbaum and Bowman’s method), significant difference from the corresponding negative controls

Representative images of MNed cells collected from the formalin-fixed glandular stomach, colon, and liver of rats in the treatment group are shown in Fig. 2. The GI tract MN test results of Exps.1 and 2 are shown in Fig.3A and 3B, respectively. Figure 3A shows the MNed cell frequencies obtained using the FF method compared with those obtained using the EDTA method. Both the glandular stomach and colon showed similar frequencies, with no statistically significant difference between the two methods. The treatment groups showed significant increases in the frequencies compared to the corresponding negative controls. Figure 3B summarizes the aging effect of storage in formalin fixative for at least 1 year. Both the glandular stomach and colon showed similar frequencies, with no significant difference between the initial values and values 1 year later. The treatment groups showed significant increases in the frequencies compared to the corresponding negative controls.

As for the results of the liver MN test simultaneously conducted with the GI tract MN test, significant increases in the frequency of MNed hepatocytes [mean ± SD (%): 0.61 ± 0.25 in Exp.1 and 0.51 ± 0.04 in Exp.2] were observed in the treatment groups compared to the negative controls [mean ± SD (%): 0.03 ± 0.03 in Exp.1 and 0.02 ± 0.03 in Exp.2].

In the BM MN test, significant increases in the frequency of MNed IMEs [mean ± SD (%): 1.05 ± 0.48 in Exp.1 and 0.66 ± 0.24 in Exp.2] were also observed in the treatment groups compared to the negative controls [mean ± SD (%): 0.13 ± 0.06 in Exp.1 and 0.06 ± 0.07 in Exp.2]. There was no significant difference in %IME values between the treatment [mean ± SD (%): 43.66 ± 7.09 in Exp.1 and 44.08 ± 5.86 in Exp.2] and negative control groups [mean ± SD (%): 49.86 ± 3.21 in Exp.1 and 43.80 ± 8.01 in Exp.2].

**Fig. 4:**
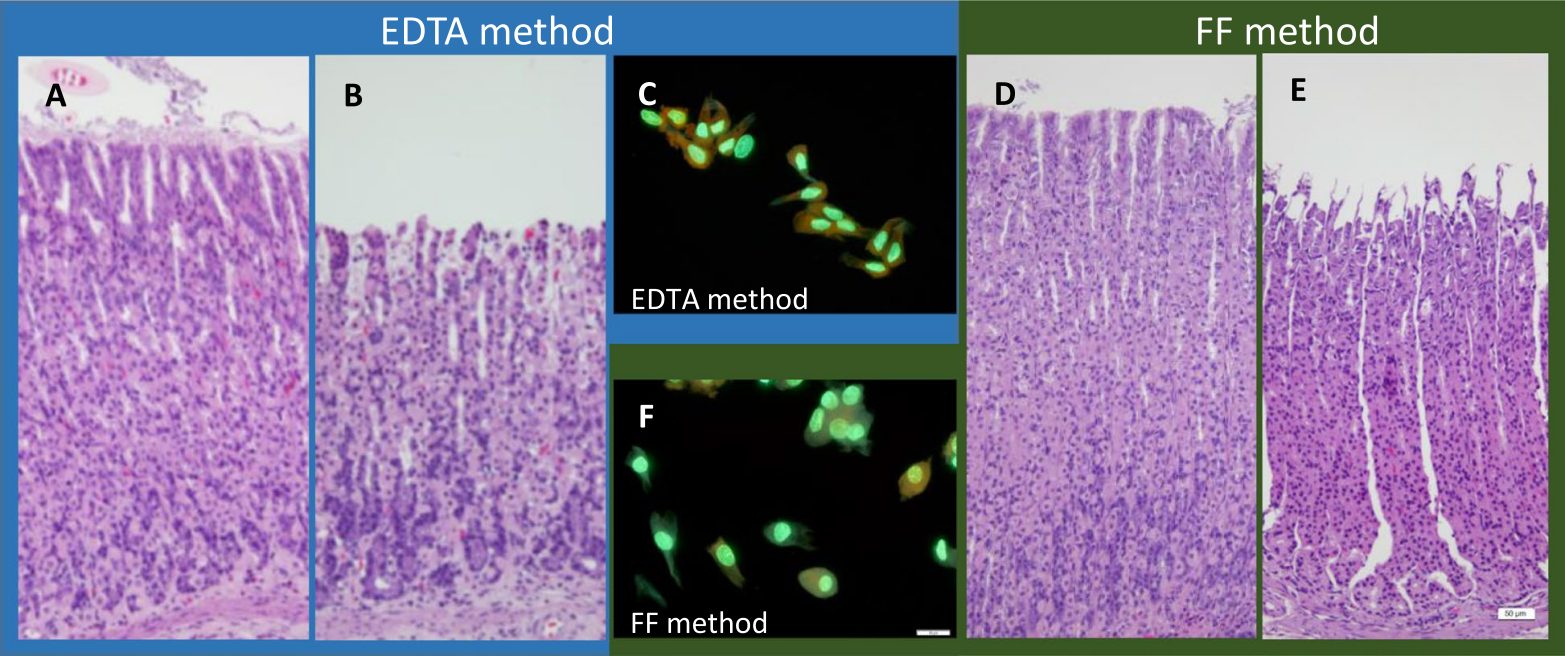
Photos of the glandular stomach before and after cell isolation and isolated cells. Histological sections before **A** and after **B** cell isolation by the EDTA method and before **D** and after **E** by FF method. The fluorescence microscopic images of the isolated cells by EDTA method **C** and of those by the FF method **F**. Bars represent 50 μm for the histological section and 20 μm for the isolated cells

**Fig. 5:**
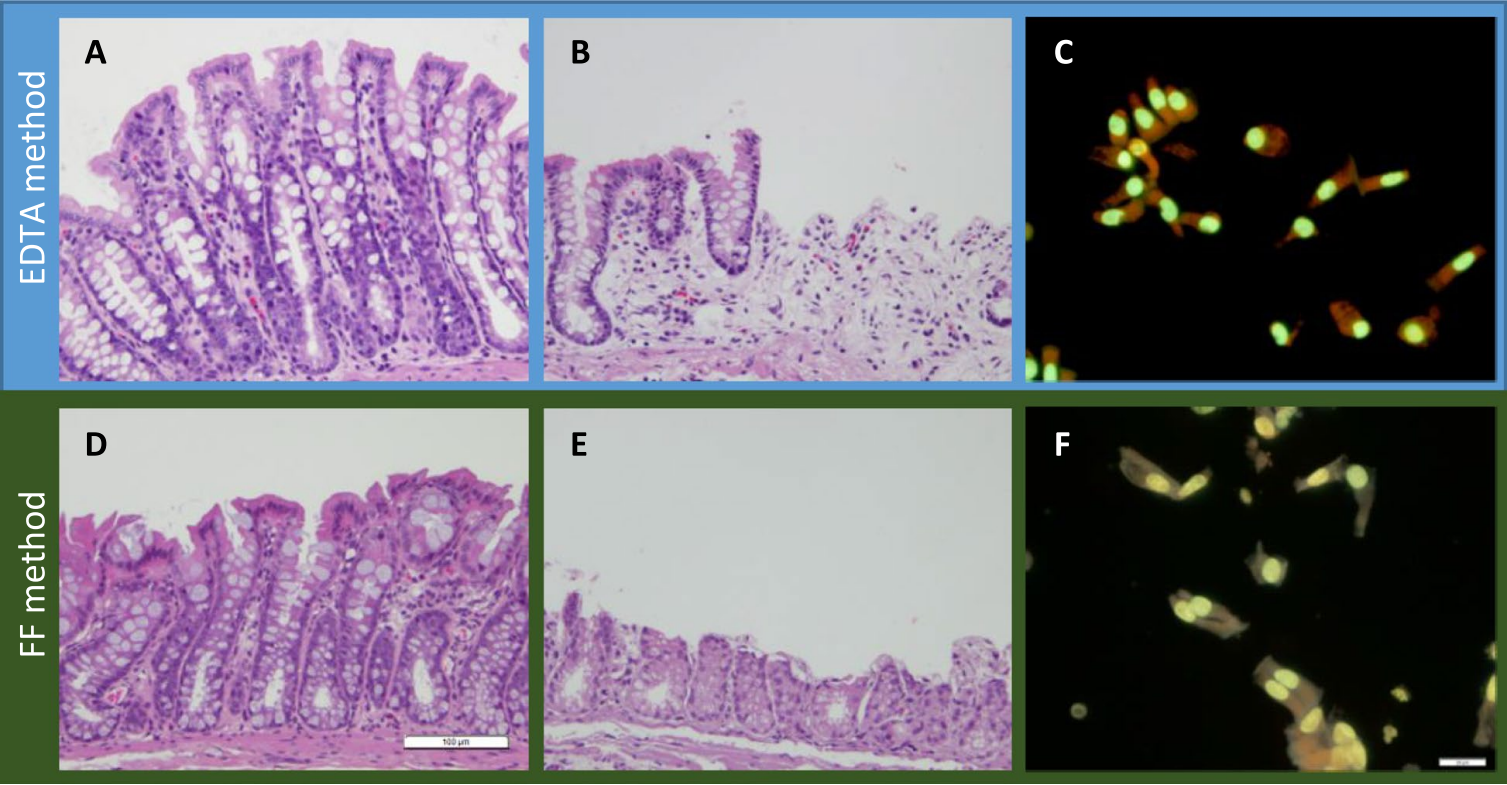
Photos of the colon before and after cell isolation and isolated cells. Histological sections before **A** and after **B** cell isolation by EDTA method and before **D** and after **E** by FF method. The fluorescence microscopic images of the isolated cells by the EDTA method **C** and of those by the FF method **F**. Bars represent 100 μm for the histological section and 20 μm for the isolated cells

According to the histological examination, the glandular stomach showed similar images with the EDTA and FF methods both before and after isolation of cells (Fig. 4A, B, D, and E). Cells in or above the neck zone (proliferative zone) of the gastric glands were collected, and they were similar in shape for both methods (Fig. 4C and F). In addition to the glandular stomach, cells in or above the proliferative zone located in the lower half of the crypts of the colon were collected (Fig. 5A, B, D, and E), and they were similar in shape for both methods (Fig. 5C and F).

## Discussion

Clastogenicity/aneugenicity evaluation using GI tract cells is important for risk assessment, because such tissues are the first contact tissues when chemicals are introduced into the body through oral exposure. Previously, we successfully developed an MN test method using EDTA for a freshly dissected glandular stomach and colon [10–13, 19]. Nevertheless, this method is tedious and time-consuming; not many samples can be processed simultaneously. Thus, further development of this method is desired. Hamada et al. [9, 20] developed a liver MN test using formalin-fixed tissue. We applied this method to the GI tract, and optimized the test conditions, such as KOH treatment and cell staining. As a result, the expected data could be obtained using the model chemicals that we used for the EDTA method as positive controls.

Our data showed good reproducibility between experiments, between the EDTA and FF methods, and between freshly prepared samples and 1-year old samples (stored in the neutral formalin fixative). Epithelial cells of the glandular stomach and colon are known to divide at the proliferative zone (neck zone in the gastric gland and lower half of the colonic crypt) [21], and the divided cells migrate upward to the luminal surface in 48–96 h [19]. The present study showed that both methods could be used for collecting cells located in or above the proliferative zone of the GI tract tissues, in which the divided cells after chemical treatment would be included. The MN test results were comparable between the two methods. Therefore, the equivalence of these methods was confirmed.

Compared to the EDTA method using fresh tissues, this improved method using formalin-fixed tissues not only allows easier isolation of cells from the GI tract tissues but also makes it much easier to share the tissues for histopathological examination when incorporating the GI tract MN test into a general toxicity study. Furthermore, the FF method can be applied to formalin-fixed samples from other toxicological studies retrospectively when needed, as well as the repeated-dose liver MN test [20]. Histological sections could also be used to investigate cell proliferation in the GI tract tissues of the same animals used for MN analysis, as shown in our previous reports using Ki-67 immunohistochemistry [10–13]. Accordingly, we do not have to perform new animal experiments only for the GI tract MN test. Overall, the FF method is ideal for supporting animal welfare as it reduces the use of experimental animals.

## Conclusion

The equivalence of the EDTA and FF methods was confirmed, and MN analysis was possible up to at least 1 year after formalin fixation of the GI tract, indicating that the FF method is valuable for the GI tract MN test.

## Data Availability

All data generated or analyzed during this study are included in this published article.

## Abbreviations

MN: micronucleus
GI: gastrointestinal
FF method: formalin-fixation method
DEN: diethylnitrosamine
DMH: 1,2-dimethylhydrazine dihydrochloride
